# Comparative Findings Between ^68^Ga-PSMA and ^18^F-FDG PET/CT for Hepatocellular Carcinoma

**DOI:** 10.4274/mirt.galenos.2020.50455

**Published:** 2020-10-19

**Authors:** Seval Erhamamcı, Nesrin Aslan

**Affiliations:** 1 Başkent University Faculty of Medicine, Ankara; Başkent University İstanbul Hospital, Department of Nuclear Medicine, İstanbul, Turkey; 2 Neolife Medical Center, Clinic of Nuclear Medicine, İstanbul, Turkey

**Keywords:** Hepatocellular carcinoma, bone metastases, PET/CT, 68Ga-PSMA, 18F-FDG

## Abstract

We have reported here the case of a 69-year-old man who presented with spinal cord compression due to bone metastases as the first manifestation of hepatocellular carcinoma (HCC). For the initial staging, the patient underwent ^18^F-fluorodeoxyglucose (FDG) positron emission tomography/computerized tomography (PET/CT) imaging, which demonstrated mild ^18^F-FDG uptake in the multiple expansile osteolytic bone lesions, but no remarkable atypical ^18^F-FDG uptake in the liver lesion on low-doses CT. An additional PET/CT scan was performed to evaluate the prostate-specific membrane antigen (PSMA) expression, which has recently been reported to be a potential biological marker in a variety of tumors including HCC. High PSMA uptake was recorded in both the metastatic bone lesions and the primary liver lesion/tumor by the ^68^Ga-PSMA PET/CT.

## Abstract (tr)

Hepatoselüler karsinomun (HSK) ilk belirtisi olarak, kemik metastazının neden olduğu spinal kord kompresyonu ile başvuran 69 yaşında bir erkek hastayı tanımladık. Başlangıç evreleme için, hastaya ^18^F-florodeoksiglukoz (FDG) pozitron emisyon tomografisi/bilgisayarlı tomografi (PET/BT) görüntüleme yapıldı. Görüntüleme çok sayıda ekspansil osteolitik kemik lezyonlarında hafif ^18^F-FDG tutulumu gösterdi ve düşük doz BT’deki karaciğer lezyonunda kayda değer atipik ^18^F-FDG tutulumu göstermedi. HSK’da dahil olmak üzere çeşitli tümörlerde potansiyel bir biyolojik marker olduğu bildirilen prostat spesifik membran antijen (PSMA) ekspresyonunu değerlendirmek için ek bir PET/BT görüntüleme yapıldı. Hem metastatik kemik lezyonlarında hem de primer karaciğer lezyonunda/tümöründe yüksek PSMA tutulumu ^68^Ga-PSMA PET/BT ile saptandı.

## Figures and Tables

**Figure 1:**
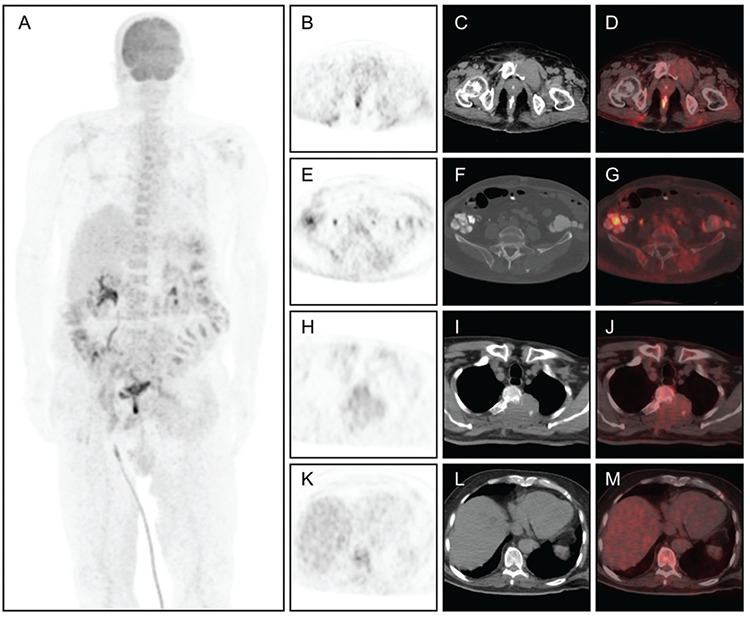
The case patient was a 69-year-old man who presented with the complaint of back pain. His magnetic resonance imaging revealed multiple metastatic lesions of the thoracic vertebral with spinal cord compression and bilateral iliac bones. Excisional biopsy of the T2-3 vertebral lesion due to spinal cord compression was also performed. Histopathological examination demonstrated metastatic malign tumor, which was consistent with the signs of hepatocellular carcinoma (HCC) metastases. The patient accordingly underwent ^18^F- fluorodeoxyglucose (FDG) positron emission tomography/computerized tomography (PET/CT) imaging for initial staging. The scan MIP **(A)**, transaxial PET **(B, E, H, K)**, **CT** **(C, F, I, L)**, and fused **(D, G, J, M)** images revealed mild uptake [maximum standardized uptake value (SUV_max_)= 4.8] in the multiple osteolytic bone lesions in the thoracal vertebra, iliac bones, and sacroiliac joints, most of which also showed remarkable soft tissue components. On the other hand, no significant atypical uptake was noted in the primary liver tumor in the corresponding low-dose CT

**Figure 2:**
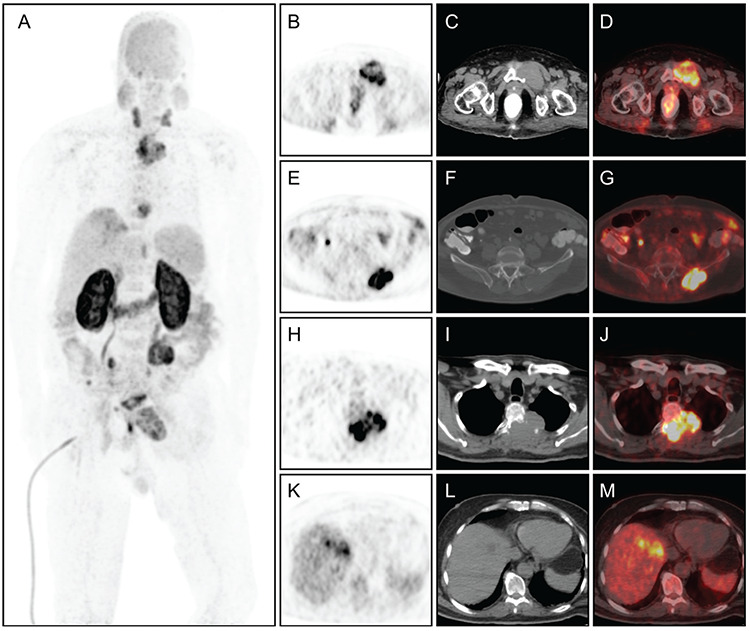
As an alternative PET/CT imaging, ^68^Ga-prostate-specific membrane antigen (PSMA) PET/CT was performed the same day. Corresponding to the lesions in ^18^F-FDG PET, ^68^Ga-PSMA PET/CT MIP **(A)**, transaxial PET **(B, E, H, K)**, CT **(C, F, I, L)**, and fused **(D, G, J, M)** images demonstrated high PSMA expression in the metastatic bone lesions (SUV_max_=23.9) and in the primary liver tumor (SUV_max_=12.1). No other findings showed metastatic disease elsewhere in the body. PET/CT imaging with ^18^F-FDG has low diagnostic accuracy in assessing HCC patients because of its low metabolism (1). ^68^Ga-PSMA PET/CT is a new diagnostic technique to image recurrent prostate cancer (2). However, increased PSMA expression has been reported for different non-prostate malignancies, including HCC (3,4,5,6,7,8). There are only a few published documents on the merits of PSMA-PET for HCC (3,4,5,6,7,8). In fact, a few case reports and only 2 studies involving a small sample size has been reported in the recent past. In one of these studies, ^68^Ga-PSMA was reported to be superior relative to ^18^F-FDG for imaging HCC patients (7). However, in another study on advanced HCC patients, the PSMA expression was detected by ^68^Ga-PSMA PET, but it was not superior to that by ^18^F-FDG PET (8). In the current case, ^68^Ga-PSMA uptake was extremely high as compared to ^18^F-FDG uptake for bone metastases, and without ^18^F-FDG uptake in the primary tumor. Therefore, it is suggested that PET imaging with ^68^Ga-PSMA is helpful in HCC patients with low FDG affinity. Moreover, we believe that the existence of PSMA expression may act as a guide for radioligand therapy targeting PSMA in the future
